# Ambulatory care after acute kidney injury: an opportunity to improve patient outcomes

**DOI:** 10.1186/s40697-015-0071-8

**Published:** 2015-10-06

**Authors:** Samuel A. Silver, Stuart L. Goldstein, Ziv Harel, Andrea Harvey, Elizabeth J. Rompies, Neill K. Adhikari, Rey Acedillo, Arsh K. Jain, Robert Richardson, Christopher T. Chan, Glenn M. Chertow, Chaim M. Bell, Ron Wald

**Affiliations:** Division of Nephrology, St. Michael’s Hospital, University of Toronto, Toronto, Canada; Center for Acute Care Nephrology, Cincinnati Children’s Hospital, Cincinnati, OH USA; Department of Medicine and Keenan Research Center, Li Ka Shing Knowledge Institute of St Michael’s Hospital, University of Toronto, Toronto, Canada; Department of Critical Care Medicine, Sunnybrook Health Sciences Center, University of Toronto, Toronto, Canada; Division of Nephrology, Western University, London, Canada; Division of Nephrology, University Health Network-Toronto General Hospital, University of Toronto, Toronto, Canada; Divison of Nephrology, Stanford University School of Medicine, Palo Alto, CA USA; Institute of Health Policy, Management, and Evaluation, University of Toronto, Toronto, Canada; Department of Medicine, Mount Sinai Hospital, University of Toronto, Toronto, Canada; Present Address: 190 King Edward Avenue, Toronto, ON M4C 5J8 Canada

## Abstract

**Purpose of review:**

Acute kidney injury (AKI) is an increasingly common problem among hospitalized patients. Patients who survive an AKI-associated hospitalization are at higher risk of *de novo* and worsening chronic kidney disease, end-stage kidney disease, cardiovascular disease, and death. For hospitalized patients with dialysis-requiring AKI, outpatient follow-up with a nephrologist within 90 days of hospital discharge has been associated with enhanced survival. However, most patients who survive an AKI episode do not receive any follow-up nephrology care. This narrative review describes the experience of two new clinical programs to care for AKI patients after hospital discharge: the Acute Kidney Injury Follow-up Clinic for adults (St. Michael’s Hospital and University Health Network, Toronto, Canada) and the AKI Survivor Clinic for children (Cincinnati Children’s Hospital, USA).

**Sources of information:**

MEDLINE, PubMed, ISI Web of Science

**Findings:**

These two ambulatory clinics have been in existence for close to two (adult) and four (pediatric) years, and were developed separately and independently in different populations and health systems. The components of both clinics are described, including the target population, referral process, medical interventions, patient education activities, and follow-up schedule. Common elements include targeting patients with KDIGO stage 2 or 3 AKI, regular audits of the inpatient nephrology census to track eligible patients, medication reconciliation, and education on the long-term consequences of AKI.

**Limitations:**

Despite the theoretical benefits of post-AKI follow-up and the clinic components described, there is no high quality evidence to prove that the interventions implemented in these clinics will reduce morbidity or mortality. Therefore, we also present a plan to evaluate the adult AKI Follow-up Clinic in order to determine if it can improve clinical outcomes compared to patients with AKI who do not receive follow-up care.

**Implications:**

Follow-up of AKI survivors is low, and this review describes two different clinics that care for patients who survive an AKI episode. We believe that sharing the experiences of the AKI Follow-up Clinic and AKI Survivor Clinic provide physicians with a feasible framework to implement their own clinics, which may help AKI patients receive outpatient care commensurate with their high risk status.

**Electronic supplementary material:**

The online version of this article (doi:10.1186/s40697-015-0071-8) contains supplementary material, which is available to authorized users.

## ABRÉGÉ

**Objectifs de la revue:**

L’insuffisance rénale aiguë (IRA) est un problème de plus en plus fréquent chez les patients hospitalisés. Ceux d’entre eux qui survivent à un épisode d’IRA courent un risque élevé de développer une insuffisance rénale chronique et des maladies cardiovasculaires ou d’aggraver leur état vers l’insuffisance rénale terminale et donc, la mort. Chez les patients hospitalisés pour insuffisance rénale aiguë nécessitant une hémodialyse, un taux de survie accru a été associé à un suivi par un néphrologue dans les 90 jours suivant la sortie de l’hôpital. Cependant, la majorité de ces patients ne bénéficient d’aucun suivi en néphrologie post-hospitalisation. La présente revue narrative s’attarde à décrire l’expérience de deux nouveaux programmes cliniques qui prennent en charge les patients après leur sortie de l’hôpital à la suite d’un épisode d’IRA. Il s’agit des programmes de l’« Acute Kidney Injury Follow-up Clinic » des hôpitaux St. Micheal et UHN de Toronto, et de la clinique pédiatrique « AKI Survivor Clinic for children » de l’hôpital pour enfants de Cincinnati, aux États-Unis.

**Sources:**

MEDLINE, PubMed, ISI le web des sciences

**Constatations:**

Ces cliniques ambulatoires, qui existent depuis près de deux ans (clinique pour adultes) et de quatre ans (clinique pédiatrique) se sont développées séparément et de façon indépendante pour des populations différentes, dans des systèmes de santé différents. Les constituants de ces deux cliniques se distinguent par l’identification de la population ciblée, le processus de présentation, les interventions médicales, les activités de sensibilisation auprès des patients et leur calendrier de suivi. Les parties communes incluent le recrutement de patients atteints d’IRA de stades 2 et 3 quant aux chances de l’amélioration globale de leurs résultats (KDIGO). Ce recrutement se fait par le passage en revue à intervalles réguliers des listes de patients hospitalisés aux unités néphrologiques afin d’identifier ceux qui sont admissibles au suivi. Ces parties communes incluent aussi un bilan comparatif des médicaments et la transmission d’informations sur les conséquences à long terme de l’insuffisance rénale aiguë.

**Limites de l’étude:**

Malgré les bienfaits escomptés d’un suivi post-IRA et, les éléments cliniques décrits plus haut, il n’existe aucune preuve concrète que les procédures mises en œuvre dans ces programmes réduiront la morbidité ou la mortalité associées à la maladie. Par conséquent, nous présentons également un plan d’évaluation de la clinique pour adultes en suivi post-IRA afin de déterminer si son programme peut améliorer les résultats cliniques de ses patients en comparaison avec les patients ne recevant pas ce suivi.

**Conséquences:**

De façon générale, peu de patients ayant survécu à un épisode d’IRA reçoivent un suivi adéquat post-hospitalisation. Cette revue présente deux cliniques offrant ce suivi. Nous pensons que le partage de leurs expériences respectives peut poser un cadre réalisable aux médecins traitants qui souhaiteraient mettre sur pied leurs propres cliniques, afin d’offrir à leurs patients un suivi en externe répondant adéquatement à leur état de santé précaire.

## What was known before

Patients who survive acute kidney injury are at increased risk for chronic kidney disease and death. However, less than 20 % of patients see a nephrologist within 3 months of hospital discharge, even though early nephrology follow-up after acute kidney injury has been associated with enhanced survival.

## What this adds

The description of two different and independently developed clinical programs to care for AKI patients after hospital discharge: the Acute Kidney Injury Follow-up Clinic for adults (St. Michael’s Hospital and University Health Network, Toronto, Canada) and the AKI Survivor Clinic for children (Cincinnati Children’s Hospital, USA). These clinics have been operating for close to two and four years respectively, and their experiences provide a framework for other centers to implement similar programs for AKI survivors.

## Background

Acute kidney injury (AKI) is an abrupt deterioration in kidney function that complicates 15–20 % of all hospital stays and is the most frequent reason for inpatient nephrology consultation [1]. Due in part to the aging population, the incidence of AKI is increasing and is expected to double over the next decade [2].

Although the in-hospital outcomes of AKI are ominous, it has become increasingly clear that individuals who survive to leave the hospital after an episode of AKI are at persistent risk of adverse outcomes. A recent meta-analysis demonstrated that compared to patients without AKI, patients who survive an AKI episode have an almost ten-fold higher risk of developing *de novo* chronic kidney disease (CKD), a three-fold higher risk of end-stage renal disease (ESRD), and twice the risk of death [3]. A cohort study also demonstrated a 67 % higher risk of cardiac events in survivors of AKI requiring dialysis [4]. In addition, patients who survive AKI have worse long-term outcomes than patients with diabetes mellitus and survivors of an ST-elevation myocardial infarction [4, 5].

While the nature of the relation (causal versus correlative) between AKI and downstream adverse events remains a topic of debate [6, 7], there is no doubt that for most patients, an episode of AKI heralds an ominous prognosis. We agree with the American Society of Nephrology AKI Advisory Group and other experts that proof of causality is not necessary for action to improve the quality of care post-AKI [2, 8]. The provision of more intensive post-AKI outpatient care has the potential to identify early evolution to CKD and to mitigate complications and costs associated with CKD progression [9, 10]. Unfortunately, the majority of patients who survive AKI are not seen by a nephrologist upon hospital discharge. Even among patients hospitalized with dialysis-requiring AKI (the most extreme form of AKI) who recover sufficient kidney function to no longer require dialysis, fewer than 50 % see a nephrologist within one year of hospital discharge based upon data from the United States Renal Data System [11]. At the very least, the strong association between AKI and adverse events warrants the same degree of attention as other high risk populations. For example, cardiac care does not cease with the initial hospitalization for a myocardial infarction or congestive heart failure. Rather, such sentinel events initiate a long-term relationship with a cardiologist, whose care is focused on secondary prevention strategies.

### Poor follow-up of after an AKI episode: Scope of the problem

Few studies have measured AKI outpatient follow-up rates in a standardized manner. A single-center study in Scotland examined the records of all patients (inpatients and outpatients) with a serum creatinine ≥ 300 μmol/L over a one year period performed at their hospital laboratory [12]. AKI was defined as a new rise in serum creatinine ≥ 300 μmol/L, but no definition of baseline creatinine was provided. Patients were excluded if they had a pre-existing serum creatinine ≥ 200 μmol/L or were dialysis-dependent more than 90 days after the AKI episode. Of the 310 AKI patients identified, 22 % (70/310) were referred to a nephrologist, or 34 % after patients with advanced cancer and persons over 80 years of age were excluded. No analysis was performed to control for the competing risk of death. Factors associated with non-referral included older age, multimorbidity, volume depletion, and cardiogenic shock. The only observed benefit from nephrology referral was amongst elderly patients with comorbidities, where nephrology referral was associated with reduced mortality compared to similar patients who were not referred to a nephrologist.

Siew et al. examined the likelihood of nephrology referral among patients with AKI in the United States Department of Veterans Affairs database [13]. Patients were excluded if they had a baseline estimated glomerular filtration rate (eGFR) ˂ 15 mL/min/1.73 m^2^ or recovered kidney function to eGFR > 60 mL/min/1.73 m^2^ within 30 days of their peak serum creatinine concentration. The Acute Kidney Injury Network (AKIN) classification system was used to stage AKI episodes, and 87 % of patients experienced AKIN stage I injury. 11 % of patients were referred to a nephrologist within 30 days. Excluding these early referrals, the proportion of patients who were referred to a nephrologist before dying, initiating dialysis, or experiencing an improvement in kidney function was 4 % at three months and 9 % at one year. Non-referred patients tended to be slightly older, were less likely to have a diagnosis of diabetes mellitus or congestive heart failure, and had a modestly higher eGFR at baseline. At the end of the one year study, 36 % of the cohort had an eGFR (eGFR) ˂ 60 mL/min/1.73 m^2^ without having been referred to a nephrologist. In patients with a baseline eGFR ≥ 60 mL/min/1.73 m^2^, 50 % recovered and 50 % had persistent dysfunction (2 % with eGFR < 30 mL/min/1.73 m^2^) at one year after the AKI episode. In patients with a baseline eGFR < 60 mL/min/1.73 m^2^, 50 % had an eGFR ≥ 45 mL/min/1.73 m^2^, 40 % had an eGFR between 30 and 44 mL/min/1.73 m^2^, and 10 % had an eGFR < 30 mL/min/1.73 m^2^ at one year after the AKI episode. It is important to note that this study only measured nephrologist referrals, with no assessment of the patient’s attendance at an appointment.

Harel et al. determined the association between nephrology follow-up within 90 days of dialysis-requiring AKI and survival using Ontario-wide administrative healthcare databases from the Institute for Clinical Evaluative Sciences (ICES) [14]. Propensity scores were used to match patients with and without nephrology follow-up. Only 41 % of patients visited a nephrologist within 90 days of discharge. Patients with pre-existing CKD and prior nephrologist visits were more likely to receive post-AKI follow-up. Nephrology follow-up was associated with a 24 % lower risk of death at two years compared to patients who did not receive nephrology follow-up.

In summary, the majority of patients who survive AKI, even those with the most severe forms, do not receive specialized nephrology care after hospital discharge. Given the rising incidence of AKI and the poor outcomes associated with it, an intervention to mitigate the risks of such outcomes may have a significant public health impact.

This narrative review describes two ambulatory care clinics for AKI survivors that were developed separately and independently, and have each been in existence for close to two (adult) and four (pediatric) years. We outline the components of the two different clinics: the AKI Follow-up Clinic for adults at St. Michael’s Hospital and the University Health Network in Toronto, Ontario, Canada, and the AKI Survivor Clinic for children at the Center for Acute Care Nephrology at Cincinnati Children’s Hospital Medical Center in Cincinnati, Ohio, USA. We will also describe current plans to evaluate the effect of the adult AKI Follow-up Clinic model on clinical outcomes.

## Components of the AKI Follow-Up Clinic for Adults

The following section describes the elements of an AKI Follow-up Clinic for adults that are currently utilized at St. Michael’s Hospital and the University Health Network in Toronto, Ontario, Canada. The St. Michael’s clinic has been operating since September 2013, and the University Health Network clinic has been operating since October 2014. These clinics have assessed 150 and 65 new AKI patients since their respective introductions.

### Target population

We utilize the following AKI Follow-up Clinic referral criteria.

### Inclusion

- Kidney Disease Improving Global Outcomes (KDIGO) stage 2 AKI and above (including need for dialysis) [15].

### Exclusion

- Kidney transplant recipients.
- Baseline eGFR under 30 mL/min/1.73 m^2^.
- Diagnosis of: glomerulonephritis, vasculitis with kidney involvement, hemolytic-uremic syndrome, polycystic kidney disease, multiple myeloma.
- Palliation as primary goal of care.
- Patients with previously established and ongoing nephrology follow-up, including patients discharged with a persistent requirement for renal replacement therapy.

### Rationale

AKI severity appears to be the most important risk factor for adverse post-discharge outcomes, [16, 17]. The unanswered question is what threshold of AKI warrants follow-up. Some experts have advocated that nephrology follow-up occur for all patients with KDIGO stage 2–3 AKI [2]. However, the association with CKD, ESRD, and mortality seems to be present even among patients with mild and rapidly reversible AKI who are discharged from hospital with normal or near normal kidney function [18].

Ideally, a simple and practical risk score would identify patients at high risk for CKD progression and mortality post-AKI. These patients could then be selectively targeted for early nephrologist follow-up, as they would be most likely to benefit. Previous studies of patients who survive AKI have reported predictors of kidney disease progression and mortality [19, 20, 16, 21–23]. While there are notable differences in methodology, case-mix, and outcome ascertainment, many of these studies identified similar risk factors. These include: previous nephrology consultation, a history of CKD, pre-existing hypertension or cardiovascular disease, older age, recurrent AKI, and higher serum creatinine one year post-AKI. One study combined several risk factors into a score, but its feasibility is limited by the inclusion of serum albumin, a laboratory parameter that may not be routinely measured in the outpatient setting [16]. Until a post-AKI risk score is developed and tested under real-life conditions, our AKI Follow-up Clinic will target all patients with KDIGO stage 2–3 AKI. The reasons we have chosen to focus on this patient population are twofold: 1) patients with KDIGO stage 2–3 AKI are at greatest risk for adverse events and so most likely to benefit from nephrologist follow-up and 2) concern that including KDIGO stage 1 AKI patients would exceed the current capacity of our outpatient nephrology clinics. This was a local decision based upon our AKI Follow-up Clinic volumes and capacity, and centers with greater or fewer outpatient resources are encouraged to determine their own clinic criteria until more evidence becomes available.

There are some patients with AKI for whom alternate settings of post-discharge follow-up might be more appropriate. Patients with a baseline eGFR under 30 mL/min/1.73 m^2^ would either have already seen a nephrologist, or would benefit from a multidisciplinary clinic with a focus on dialysis planning given the frequent need for renal replacement therapy in this sub-population [24]. Therefore, we redirect these patients to a CKD clinic rather than an AKI Follow-up Clinic. Some parenchymal kidney diseases (glomerulonephritis, vasculitis with kidney involvement, hemolytic-uremic syndrome, polycystic kidney disease, multiple myeloma) would necessitate monitoring and therapies that are better served by a general nephrology clinic. In our experience, most of these patients already have nephrology follow-up arranged at the time of hospital discharge, but we clarify unclear situations with the inpatient medical team if needed. Lastly, an AKI Follow-up Clinic is redundant for patients with established and ongoing nephrology follow-up (including kidney transplant recipients), and we inform such patients to arrange an appointment with their current nephrologist shortly after hospital discharge.

### Referral process and appointment targets

**Fig. 1:**
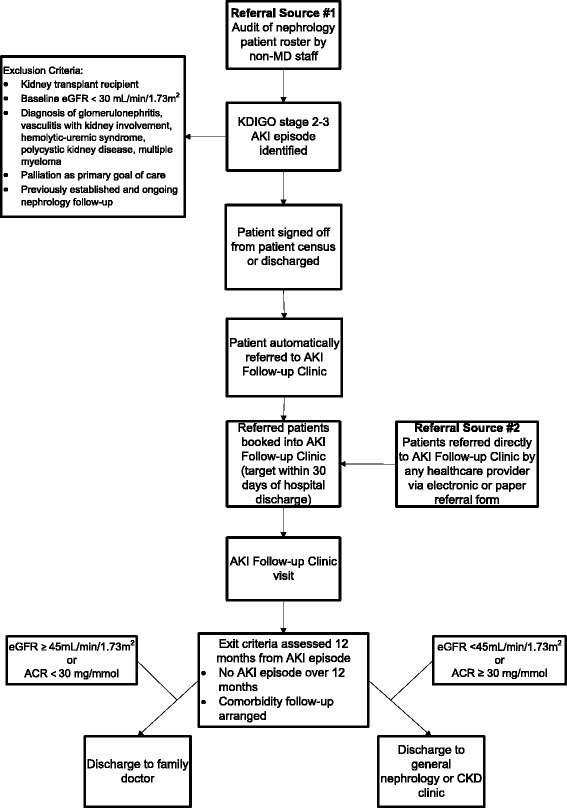
Acute Kidney Injury Follow-up Clinic referral process (adult clinic). Legend: ACR = urine albumin to creatinine ratio, AKI = acute kidney injury, CKD = chronic kidney disease, eGFR = estimated glomerular filtration rate, KDIGO = kidney disease improving global outcomes

We utilize the following referral process and appointment targets (Fig. 1).

- Referrals accepted from all hospital units through an electronic or paper referral form (Additional file 1: Figure S1)
- Weekly audits by AKI Follow-up Clinic staff to identify patients with AKI who are not referred to clinic at hospital discharge
- Target AKI Follow-up Clinic appointments within 30 days of hospital discharge

### Rationale

The first step in designing a referral process is to identify the reasons for low follow-up rates after an episode of AKI. To accomplish this objective, we first created a stakeholder map and engaged leaders of key stakeholder groups (nephrologists, cardiovascular surgeons, general internists, AKI researchers, trainees, nurse practitioners, social workers, and hospital administrators) via electronic mail. Each stakeholder group then nominated a member to join the quality improvement team, which communicated either in-person or electronically at three month intervals. These representatives identified the following as the most important local reasons for low follow-up rates after AKI: 1) lack of appreciation of the importance of AKI follow-up; 2) competing health problems in patients with AKI that are deemed higher priority during both the inpatient and outpatient period; 3) long hospital stays with hospital discharge occurring after AKI has resolved; and 4) multiple healthcare providers per AKI admission and frequent handovers, with lack of perceived responsibility for managing AKI follow-up.

With these challenges in mind, we created an online referral form (Additional file 1: Figure S1) and educational posters (Additional file 2: Figure S2) to facilitate referrals throughout the hospital. The poster is located at nurse and physician work stations on high volume AKI wards (nephrology, cardiovascular surgery, general internal medicine, critical care unit), visible only to healthcare professionals. In addition, we have an administrative or research assistant closely monitor the nephrology consult service patient roster to identify eligible patients and track these patients through their hospital stay even after the nephrology team’s involvement has ceased. This ensures that such patients are referred to the AKI Follow-up Clinic at the time of hospital discharge. This process has been in place since the inception of the AKI Follow-up Clinic. We review its operation on a weekly basis after each clinic, including the staff time required. On average, the screening process requires two hours of staff member time per week, with slight fluctuations based on AKI volumes. Due to the low time commitment, the assistant’s time is funded through clinical programs at the hospital level (~$6500/year).

A major limitation of this approach is the absence of an audit system for patients who are not on the nephrology consult service patient roster. We are currently exploring solutions to this problem, which include electronic AKI surveillance mechanisms and automatic referral prompts to the healthcare team at the time of patient discharge [25–27].

A 30 day appointment target was chosen to align with other medical disciplines and hospital readmission targets set by quality improvement agencies and insurers, including the Centers for Medicare and Medicaid Services [28–30]. Though a first appointment within this time window might not always be feasible to achieve, setting a stretch aim is a well-known quality improvement strategy to drive system change [31], and leaves room for patients to be seen by the 90 day threshold associated with decreased mortality.

### AKI Follow-up Clinic medical interventions

We utilize the following standards for each patient encounter (Additional file 3: Figure S3).

- Patient sees a nephrologist at every clinic visit
- Dedicated recommendation section to indicate medications that should be adjusted and/or stopped
- Medical therapy that is in accordance with established hypertension, diabetes, lipid, and CKD practice guidelines
- Low threshold for referral to cardiology and endocrinology for concurrent care of high risk individuals with multiple chronic diseases

### Rationale

Studies have demonstrated that nephrologists are more skilled at recognizing and managing CKD complications according to evidence-based guidelines compared with primary care providers [32, 33]. While we await the completion of randomized interventional studies on AKI patients, we cannot be sure that any medical intervention will be effective in mitigating CKD, ESRD, and death in the post-AKI setting. Nonetheless, it seems reasonable to provide simple, low cost interventions that have a plausible clinical rationale.

This list includes medication reconciliation; up to 67 % of patients admitted to the hospital have unintended medication omissions that remain common at discharge [34, 35]. Many vital medications, whose suspension in hospital might have been appropriate in light of the acute clinical circumstances, may not have been restarted by the time of discharge. These discrepancies have been associated with death and hospital readmission, particularly for cessation of chronic disease medications such as statins and anti-platelets agents [36]. In addition, patients may have new medications started in hospital, which we review to ensure proper dosing based on kidney function and clear indications to avoid polypharmacy.

Since patients who survive AKI have worse long-term outcomes than patients with diabetes and coronary artery disease, it seems reasonable to ensure they are meeting established targets recommended by hypertension, diabetes, lipid, and CKD practice guidelines [37–40]. We also mail patients a laboratory requisition prior to clinic that includes measurement of hemoglobin, electrolytes, bicarbonate, serum creatinine, calcium, phosphate, serum albumin, glucose, lipid profile, uric acid, and urine albumin to creatinine ratio.

In addition, increased referrals to specialist colleagues may lead to better management of common coexisting conditions, such as heart failure and diabetes mellitus. The merits of combined care on survival have been demonstrated in multiple disciplines, and improved access to healthcare resources may be an important mediator of downstream outcomes in AKI patients [41, 42]. For patients already followed by multiple specialists, a combined clinic approach where the patient can visit with all of their specialists in a single location may be an effective strategy to enhance follow-up of AKI survivors. Combined clinics are not a new approach to care [43], and may warrant further study for AKI survivors with multiple comorbidities.

### Patient, family and healthcare provider education

We employ the following patient, caregiver, and provider education standards.

- Inform patients at first visit that AKI is associated with accelerated CKD, ESRD, and cardiac events
- Provide patients with a “sick-day” medication list (Additional file 4: Figure S4), so that they are aware which medications to stop when feeling ill
- Send referral notes to the patient’s primary care provider and relevant specialists after each visit, educating them on the long-term prognosis of AKI

### Rationale

AKI is generally a “silent” component of a patient’s hospitalization, and often patients are not aware that they experienced an episode of AKI. At each clinic visit, we review with patients the natural history of AKI and the potential long-term consequences. This serves as a bridge to discuss medical interventions, especially cardioprotective lifestyle measures. Patients may be more motivated to adhere to lifestyle changes and medication regimens if they are made aware of the long-term consequences of AKI [44]. To promote self-care, we teach patients to hold their diuretics, angiotensin-converting-enzyme inhibitors, and angiotensin receptor blockers during episodes of intravascular volume depletion. This patient population has already declared itself as susceptible to AKI; temporarily stopping these medications is a reasonable strategy to protect against recurrent AKI.

In our opinion and based on our experience, the first AKI Follow-up Clinic visit is the optimal time to educate patients on the long-term effects of AKI, since they have started to recover from their acute illness. We have not attempted to provide patient education during the hospitalization or prior to the clinic visit (using a mailed pamphlet). This is something we are considering in the future, as it may help alleviate patient anxiety prior to the clinic visit and ensure patient engagement with the follow-up process.

The AKI Follow-up Clinic also provides nephrologists with an opportunity to educate primary care providers and specialists on AKI and its downstream complications. Some experts have suggested that an “episode of AKI” should be documented in the medical history portion of the patient’s medical record [2]. Incorporating this recommendation into daily practice will require effective knowledge translation strategies, which an AKI Follow-up Clinic is well-positioned to accomplish. All our patient dictations conclude with the same statement: “Thank you for referring your patient to the Acute Kidney Injury Follow-up Clinic. AKI survivors have a 40 % increased risk of dying in the two years after the initial hospitalization, and AKI is associated with the development of new or accelerated chronic kidney disease. We will see patients in clinic two to three times per year, and follow bloodwork quarterly. The objective of the AKI Follow-up Clinic is to reduce the long-term morbidity and mortality of AKI survivors.”

### Follow-up visits and discharge criteria

We utilize the following criteria to monitor patients who survive AKI and determine when patients can be discharged from clinic (Fig. 1).

- Patients are followed by the AKI Follow-up Clinic for a minimum of one year
- Patients complete bloodwork to monitor kidney function, electrolytes, and proteinuria at least every three months
- Patients are eligible to graduate from the AKI Follow-up Clinic provided they have had no further AKI episode over 12 months and appropriate comorbidity follow-up has been arranged
- At clinic discharge, patients are referred for general nephrology follow-up if their eGFR is under 45 mL/min/1.73 m^2^ (stage 3b CKD) or albumin:creatinine ratio over 30 mg/mmol [39]; otherwise, they are sent back to their family physician for ongoing care along with an educational information letter.

### Rationale

The majority of post-AKI adverse events appear to occur in the first three to six months following the AKI episode [18]. Therefore, six months appears to be the minimum period of time during which kidney function should be monitored, with 12 months providing more reassurance new or accelerated CKD will not be missed.

Our approach is to monitor kidney function and albuminuria at regular intervals (minimum three months) during this high-risk time period. In this way, kidney deterioration can be recognized sooner, follow-up arranged, and the necessary steps taken to preserve the remaining kidney function.

An AKI Follow-up Clinic would be unsustainable if it must follow patients for an indefinite period of time. It would also be unable to meet its appointment targets for new patients. We have established pre-specified clinic graduation criteria after one year of follow-up, which consists of no recurrent AKI episodes and appropriate comorbidity follow-up. Patients are transitioned to a general nephrology or CKD clinic according to evidence-based CKD guidelines [39]. The remaining patients return to the care of their family physician, and are provided with an exit pamphlet that outlines their AKI diagnosis, yearly kidney monitoring, and instructions on re-referral for nephrology care (Additional file 5: Figure S5). Long-term patient outcomes are ascertained by linkage to Ontario-wide administrative healthcare databases from ICES, for which patient consent is obtained at the first clinic visit. This creates an AKI survivor database for future research.

Thus far, this discharge criteria has helped the AKI Follow-up Clinic to maintain a mean appointment time of 30 days from hospital discharge. This has been accomplished using a weekly half-day clinic model at a tertiary center with 75 new AKI survivor assessments per year. Until more evidence on follow-up duration after AKI is available, programs are encouraged to modify the discharge criteria to suit local outpatient models, AKI volumes, and care practices.

## Components of the AKI survivor clinic for children

The following section describes the elements of the AKI Survivor Clinic for children that are currently utilized at the Cincinnati Children’s Hospital, a tertiary care pediatric hospital in the United States. This clinic and its components were devised separately and independently from the adult clinics that were previously described. This children’s clinic has been operating since July 2011 and is available to all hospitalized children from the intensive care units and non-critical care wards who meet the AKI criteria below. The AKI Survivor Clinic has assessed 300 children (~75 per year) with AKI since its introduction.

### Target population

The inclusion criteria for the AKI Survivor Clinic for children is similar to adults—hospitalized children who have experienced KDIGO stage 2–3 AKI for at least 48 hours, since this degree of AKI is associated with short-term morbidity and mortality [45–47]. It is not yet definitively known which patients will progress to CKD, and less is known if this progression is dependent upon the presumed source of AKI, diagnosis and concomitant factors, or a genetic component. Therefore, we have maintained broad inclusion criteria.

### Referral process and appointment targets

We have utilized our electronic health record (EPIC™, Verona, Wisconsin) to identify eligible AKI patients, with daily trigger reports stratified by the serum creatinine KDIGO AKI criteria. These AKI trigger reports are sent via an e-mail link to a Center for Acute Care Nephrology (CACN) team member. This allows the CACN to track details that include the date of the AKI episode, severity, baseline and maximum serum creatinine, patient health status, and communication with the patient’s primary medical team and/or family. Review of the trigger report and entering eligible patients into an adapted tracking tool takes 30 minutes per day, and coordination with the patient’s primary team and meeting with the family requires 30 minutes per patient. Currently, about two new patients per week are identified. This tracking tool also automatically populates AKI Survivor Clinic appointment dates at three, six, and twelve months. The tracking tool is audited twice per month, and a CACN nurse practitioner contacts the patient’s primary inpatient service, the primary care provider, or patient/family directly when a follow-up appointment is approaching. Since our center considers follow-up for severe AKI, as defined above, as standard of care, the clinic is funded by reimbursement for patient clinic visits. We have not received any payment denials since the clinic’s inception.

In the case of direct patient/family contact, this is accomplished via a standard letter. One concern with this approach is the patient/family’s lack of understanding of the AKI diagnosis, which we address by including in the letter educational material that explains AKI in lay terms and provides contact information for CACN medical staff. Since pediatric AKI is most often seen as a sequela of another primary illness or its treatment (such as stem cell transplantation, liver transplantation, cardiopulmonary bypass), we bring the AKI Survivor Clinic to the patient’s primary sub-specialty clinic. This requires close coordination with primary care providers and sub-specialists so that nephrology follow-up is scheduled on the same day and at the same location as other appointments.

Similar to our adult nephrology colleagues, we based our appointment target according to the KDIGO AKI guidelines and CKD literature, which advise an evaluation of CKD post-AKI at the 90 day mark [15, 39]. Allowing 90 days for scheduling of follow-up (outside of severe cases), may increase adherence to the visit schedule (although this has yet to be evaluated). Our rationale is that a 90 day target not only permits time for the AKI Survivor Clinic to coordinate follow-up dates with other providers, but may also allow for the patient to have some recuperation post-discharge. We minimize caregiver burden for patients with other chronic illnesses by conducting AKI Survivor Clinic visits in the same location and at the same time as their primary disease clinic (e.g., cardiology or oncology clinic). Currently, we are not systematically assessing health-related quality of life in AKI survivors.

### AKI Survivor Clinic medical interventions

Our approach is similar to that of adults, with the provision of medical therapy that is in accordance with established CKD practice guidelines. An additional consideration in children is to screen for future management plans (surgery, chemotherapy) that may place patients at high risk for recurrent AKI episodes.

### Patient, family and healthcare provider education

Since AKI has virtually no signs and symptoms, patients may believe that follow-up is less important than other conditions. We address this knowledge gap by providing education on AKI, its sources, and ideal surveillance. In our experience, parents/guardians of AKI patients are especially interested in the possibility of recovery or preservation of their child’s kidney function. To facilitate education, we utilize tools such as an AKI Knowing Note and laminated wallet cards (Additional file 6: Figure S6 and Additional file 7: Figure S7). The latter provide a handy reference for patients on common prescription and over-the-counter nephrotoxic medications, with nephrology contact information for additional questions. This is intended to facilitate real-time medication reconciliation and dose adjustments. In this way, our AKI Survivor Clinic strives to promote self-care, making education arguably the most valuable component of follow-up.

### Follow-up visits and discharge criteria

Our follow-up procedures for children are similar to adults, except with less formal graduation criteria. In fact, many children are followed in the AKI Survivor Clinic for up to five years. An additional monitoring consideration is the application of cystatin C as a marker of kidney function, since decreased muscle mass in children (over the age of two) renders serum creatinine a less sensitive measure of kidney function when used in isolation [48].

## Evaluation framework for the adult AKI Follow-up Clinic

Despite the potential benefits of the AKI Follow-up Clinic, there is no high quality evidence to prove that the interventions implemented will reduce morbidity or mortality. In fact, several follow-up clinics directed at general critical care patients have had minimal impact [49, 50]. In addition, this model of follow-up care requires staff resources, nephrologist time, and patient time to attend clinic visits; therefore, these costs should be justified by clinical benefits. As the clinic is in its early stages, success is judged using process measures. These include 1) the percentage of eligible AKI survivors seen in clinic within 90 days of discharge, 2) the percentage of eligible AKI survivors referred to clinic at discharge, and 3) the percentage of AKI Follow-up Clinic patients who receive a medication adjustment at the initial clinic visit. Over time, measures endorsed by the National Quality Forum could be integrated, such as the percentage of hypertensive patients with blood pressure below 140/90 mmHg, the percentage of diabetic patients on statins and renin-angiotensin system inhibitors, and rehospitalizations rates within 30 and 90 days of discharge [51]. However, the most important measures of success are demonstrating an impact on hard clinical outcomes.

This underscores the importance of developing and evaluating the AKI Follow-up Clinic in stages, as part of a structured research program based on the Medical Research Council framework for complex interventions [52]. This comprises a series of studies that culminates in a definitive randomized controlled trial. At each stage, the AKI Follow-up Clinic will be modified based upon knowledge generated from the previous study.

### Study 1 objective

Pilot the AKI Follow-up Clinic to ensure feasibility and identification of the population of interest.

### Study 2 objective

Perform formal qualitative evaluation with patients, caregivers, and providers to determine their understanding of AKI, its long-term consequences, and their experience with the AKI Follow-up Clinic.

### Study 3 objective

Use Ontario-wide administrative healthcare databases from ICES to determine the causes of hospital readmission and death in patients who survive an AKI episode.

### Study 4 objective

Evaluate AKI Follow-up Clinic processes of care and patient outcomes in comparison to hospitalized patients surviving an episode of AKI who are not exposed to the AKI Follow-up Clinic. We will match each AKI Follow-up Clinic patient to an AKI patient who was not exposed to the AKI Follow-up Clinic (identified from ICES databases and prospective controls collected at Sunnybrook Health Sciences Center) based on the following characteristics: baseline eGFR category (<15, 15–29, 30–59, ≥ 60 mL/min/1.73 m^2^), the receipt of dialysis on the index hospitalization, and a propensity score for the receipt of follow-up in the AKI Follow-up Clinic.

### Study 5 objective

Conduct a randomized controlled trial that compares the AKI Follow-up Clinic intervention to usual care. Patients randomized to usual care will receive a letter outlining their AKI diagnosis and the long-term risks to give to their family physician. The control group may still be referred for nephrology follow-up by their healthcare provider if desired, but these patients will see a general nephrologist and not have access to the AKI Follow-up Clinic processes. This trial is currently recruiting patients at multiple centers in Toronto, Canada (NCT02483039). This has been designed as a patient-level interventional trial instead of as a cluster randomized or stepped-wedge trial due to statistical concerns on intraclass correlation with the latter two designs that would limit the effective sample size and complicate data analysis [53].

### Primary outcome (for studies 4 and 5)

Our primary outcome is major adverse kidney events (MAKE)at one year after the AKI episode. This composite endpoint has been endorsed by the National Institute of Diabetes and Digestive and Kidney Diseases clinical workgroup to harmonize outcome reporting of interventions in AKI [54].

### Ascertainment of the components of the primary outcome (MAKE)

1. Chronic dialysis: one outpatient dialysis treatment at any time after hospital discharge
2. CKD progression (either criteria qualifies as CKD progression):
  - Incident CKD = first time eGFR is under 60 mL/min/1.73 m^2^ and stays below this value for ≥ 3 months.
  - Progressive CKD = 25 % eGFR decrease from known baseline that is <60 mL/min/1.73 m^2^, defined as the first date on which eGFR decreases to ≤75 % of the baseline eGFR and does not increase above 75 % of the baseline value for that patient for ≥ 3 months.
3. Death

### Secondary outcomes

1. Time to reach individual components of MAKE
2. MAKE and individual components evaluated as binary outcomes at 30, 90 and 365 days following randomization
3. Time to a major adverse cardiac event, defined as an admission for cerebrovascular disease, congestive heart failure, myocardial infarction, or cardiac revascularization procedure
4. Time to first rehospitalization, defined as the first readmission to hospital for any reason
5. Time to first emergency department visit, defined as the first visit for any reason
6. Time to and number of subsequent AKI episodes
7. Differences in medication use (angiotensin-converting enzyme inhibitor or angiotensin-receptor blocker, beta-adrenergic antagonist, calcium channel blocker, furosemide, hydrochlorothiazide, spironolactone, anti-platelet agents, statins)
8. Change in quality-of-life as measured by EuroQol-5D-5 L instrument (selected due to its use in other kidney studies and ability to be completed in a short timeframe [55])
9. Cost-effectiveness/cost-utility analysis

## Evaluation framework for the AKI Survivor Clinic for children

There are no current plans to formally evaluate the pediatric clinic in a manner similar to our adult colleagues. Instead, we are focused on maximizing the follow-up of eligible patients. This approach is consistent with Medical Research Council recommendations to ensure complex interventions have sufficient uptake to impact clinical outcomes [52].

Currently, we are tracking two measures to define success:

1. The percentage of patients who survive a severe AKI episode and attend an AKI Survivor Clinic appointment at 3, 6, and 12 months.
2. The percentage of AKI Survivor Clinic patients who have a complete and documented medical evaluation.
  - A complete medical evaluation is defined as: serum creatinine, serum cystatin C, urinalysis, urine albumin to creatinine ratio, and measurement of systolic and diastolic blood pressure with corresponding blood pressure percentiles for patient gender and age.

Our follow-up rate at three months is currently above 60 %, and we hope to continue to improve on this success going forward.

## Conclusions

Most of our current efforts for patients with AKI are concentrated on the period when kidney function is worsening. If the patient survives and some degree of kidney recovery occurs, the consulting nephrologist typically “signs off” and there is no routine follow-up after hospital discharge. This narrative review has described the experience of two existing models to deliver structured follow-up kidney care, the AKI Follow-up Clinic and the AKI Survivor Clinic, which care for adults and children respectively. These clinics were developed separately and independently in two different populations, countries, and healthcare systems. This suggests other centers may have success in implementing similar systems to care for AKI survivors. The components of these care models are summarized in Table 1.

**Table 1:** Components of the Acute Kidney Injury Follow-up Clinic for adults and the Acute Kidney Injury Survivor Clinic for children

	Components
Target population	Adults and Children
	• KDIGO stage 2 AKI and above
Referral process and appointment targets	Adults
	• Referrals accepted from all hospital units through an electronic or paper referral form
	• Weekly audits by clinic staff to identify AKI patients who are not referred to clinic at hospital discharge
	• Target appointments within 30 days of hospital discharge
	Children
	• Twice monthly audits of a local tracking tool to identify patients eligible for follow-up
	• Close coordination with primary care providers and sub-specialists so that nephrology follow-up is scheduled on the same day and at the same location as other appointments
	• Target appointments within 90 days of hospital discharge
Medical interventions	Adults and Children
	• Patients see a nephrologist at every clinic visit
	• Dedicated recommendation section to indicate medications that should be adjusted and/or stopped
	• Medical therapy that is in accordance with established clinical practice guidelines
	• Low threshold for referral to other specialists for concordant care of high risk individuals with multiple chronic diseases
	• Screen for future management plans (surgery, chemotherapy) that may place patients at high risk for recurrent AKI episodes
Education	Adults and Children
	• Inform patients at first visit that AKI is associated with accelerated CKD, ESRD, and cardiac events
	• Provide patients with a “sick-day” medication list or wallet card
	• Send referral notes to the patient’s primary care provider and relevant specialists after each visit, educating them on the long-term prognosis of AKI
Follow-up	Adults
	• Patients are followed for a minimum of one year
	• Patients complete laboratory investigations at least every three months
	• Patients are eligible to graduate from the AKI Follow-up Clinic provided they have had no further AKI episode over 12 months and appropriate comorbidity follow-up has been arranged
	• At clinic discharge, patients are referred for general nephrology follow-up if their eGFR is under 45 mL/min/1.73 m^2^ or urine albumin:creatinine ratio over 30 mg/mmol
	Children
	• No strict discharge criteria
	• Cystatin C is used to monitor kidney function, since decreased muscle mass in children renders serum creatinine a less sensitive measure
	• Children may be followed for up to five years after the AKI episode

*AKI* acute kidney injury, *CKD* chronic kidney disease, *eGFR* estimated glomerular filtration rate , *ESRD* end-stage renal disease , *KDIGO* kidney disease improving global outcomes

We are now well positioned to conduct further research to develop and enhance these clinic models, using both traditional and quality improvement methods. We await the results of a multi-site clinical trial (NCT02483039) in adult AKI survivors that will help determine if structured post-AKI follow-up can reduce kidney and cardiac events compared to usual care. In the meantime, physicians should consider closer outpatient follow-up for AKI survivors to ensure that they receive similar care as other high risk populations.

## Additional files

Additional file 1: Figure S1. Acute Kidney Injury Follow-up Clinic referral form (adult clinic). Legend: AKI = acute kidney injury, CRRT = continuous renal replacement therapy, CV = cardiovascular, ICU = intensive care unit, IHD = intermittent hemodialysis, NP = nurse practitioner, SLED = sustained low efficiency dialysis (PDF 280 kb) Additional file 2: Figure S2. Advertisement poster for hospital wards (adult clinic). Legend: AKI = acute kidney injury, CKD = chronic kidney disease, eGFR = estimated glomerular filtration rate, HUS = hemolytic-uremic syndrome, TTP = thrombotic thrombocytopenic purpura (DOCX 43 kb) Additional file 3: Figure S3. Standardized assessment form for new patients (adult clinic). Legend: ACEi = angiotensin-converting-enzyme inhibitor, ACR = albumin to creatinine ratio, AKI = acute kidney injury, ARB = angiotensin receptor blocker, ASA = Acetylsalicylic acid, BB = beta-blocker, BP = blood pressure, CHF = congestive heart failure, CKD = chronic kidney disease, DHP = Dihydropyridine, DM2 = diabetes mellitus type 2, eGFR = estimated glomerular filtration rate, LVEF = left ventricular ejection fraction, NSAID = non-steroidal anti-inflammatory drug, PCP = primary care provider (DOCX 20 kb) Additional file 4: Figure S4. Sick day medication list (adult clinic) (DOCX 19 kb) Additional file 5: Figure S5. Exit pamphlet for patients who graduate from clinic (adult clinic) (DOCX 13 kb) Additional file 6: Figure S6. Acute Kidney Injury Knowing Note for pediatric patients (DOCX 2488 kb) Additional file 7: Figure S7. Wallet card to alert patients to potentially nephrotoxic medications (pediatric clinic) (DOCX 129 kb)

## Abbreviations

AKI: Acute kidney injury
AKIN: Acute kidney injury network
CACN: Center for Acute Care Nephrology
CKD: Chronic kidney disease
eGFR: Estimated glomerular filtration rate
ESRD: End-stage renal disease
KDIGO: Kidney disease improving global outcomes
MAKE: Major adverse kidney event

## References

[1] Chertow GM, Burdick E, Honour M, Bonventre JV, Bates DW (2005). Acute kidney injury, mortality, length of stay, and costs in hospitalized patients. J Am Soc Nephrol.

[2] Goldstein SL, Jaber BL, Faubel S, Chawla LS (2013). AKI transition of care: a potential opportunity to detect and prevent CKD. Clin J Am Soc Nephrol.

[3] Coca SG, Singanamala S, Parikh CR (2012). Chronic kidney disease after acute kidney injury: a systematic review and meta-analysis. Kidney Int.

[4] Wu VC, Wu CH, Huang TM, Wang CY, Lai CF, Shiao CC (2014). Long-term risk of coronary events after AKI. J Am Soc Nephrol.

[5] Chawla LS, Amdur RL, Shaw AD, Faselis C, Palant CE, Kimmel PL (2014). Association between AKI and long-term renal and cardiovascular outcomes in United States veterans. Clin J Am Soc Nephrol.

[6] Hsu CY (2012). Yes, AKI truly leads to CKD. J Am Soc Nephrol.

[7] Rifkin DE, Coca SG, Kalantar-Zadeh K (2012). Does AKI truly lead to CKD?. J Am Soc Nephrol.

[8] Chawla LS, Eggers PW, Star RA, Kimmel PL (2014). Acute kidney injury and chronic kidney disease as interconnected syndromes. N Engl J Med.

[9] Smart NA, Titus TT (2011). Outcomes of early versus late nephrology referral in chronic kidney disease: A systematic review. Am J Med.

[10] Arora P, Obrador GT, Ruthazer R, Kausz AT, Meyer KB, Jenuleson CS (1999). Prevalence, predictors, and consequences of late nephrology referral at a tertiary care center. J Am Soc Nephrol.

[11] U.S. Renal Data System (2007). USRDS Annual Report 2007.

[12] Khan IH, Catto GR, Edward N, Macleod AM (1997). Acute renal failure: factors influencing nephrology referral and outcome. QJM.

[13] Siew ED, Peterson JF, Eden SK, Hung AM, Speroff T, Ikizler TA (2012). Outpatient nephrology referral rates after acute kidney injury. J Am Soc Nephrol.

[14] Harel Z, Wald R, Bargman JM, Mamdani M, Etchells E, Garg AX (2013). Nephrologist follow-up improves all-cause mortality of severe acute kidney injury survivors. Kidney Int.

[15] Kidney Disease: Improving Global Outcomes (KDIGO) Acute Kidney Injury Work Group (2012). KDIGO Clinical Practice Guideline for Acute Kidney Injury. Kidney Int Suppl.

[16] Chawla LS, Amdur RL, Amodeo S, Kimmel PL, Palant CE (2011). The severity of acute kidney injury predicts progression to chronic kidney disease. Kidney Int.

[17] Ishani A, Nelson D, Clothier B, Schult T, Nugent S, Greer N (2011). The magnitude of acute serum creatinine increase after cardiac surgery and the risk of chronic kidney disease, progression of kidney disease, and death. Arch Intern Med.

[18] Bucaloiu ID, Kirchner HL, Norfolk ER, Hartle JE, Perkins RM (2012). Increased risk of death and de novo chronic kidney disease following reversible acute kidney injury. Kidney Int.

[19] Wald R, Quinn RR, Luo J, Li P, Scales DC, Mamdani MM (2009). Chronic dialysis and death among survivors of acute kidney injury requiring dialysis. JAMA.

[20] Lo LJ, Go AS, Chertow GM, McCulloch CE, Fan D, Ordonez JD (2009). Dialysis-requiring acute renal failure increases the risk of progressive chronic kidney disease. Kidney Int.

[21] Thakar CV, Christianson A, Himmelfarb J, Leonard AC (2011). Acute kidney injury episodes and chronic kidney disease risk in diabetes mellitus. Clin J Am Soc Nephrol.

[22] Harel Z, Bell CM, Dixon SN, McArthur E, James MT, Garg AX (2014). Predictors of progression to chronic dialysis in survivors of severe acute kidney injury: a competing risk study. BMC Nephrol.

[23] Brito GA, Balbi AL, Abrao JM, Ponce D (2012). Long-term outcome of patients followed by nephrologists after an acute tubular necrosis episode. Int J Nephrol.

[24] Hemmelgarn BR, Manns BJ, Zhang J, Tonelli M, Klarenbach S, Walsh M (2007). Association between multidisciplinary care and survival for elderly patients with chronic kidney disease. J Am Soc Nephrol.

[25] Porter CJ, Juurlink I, Bisset LH, Bavakunji R, Mehta RL, Devonald MA (2014). A real-time electronic alert to improve detection of acute kidney injury in a large teaching hospital. Nephrol Dial Transplant.

[26] Selby NM, Crowley L, Fluck RJ, McIntyre CW, Monaghan J, Lawson N (2012). Use of electronic results reporting to diagnose and monitor AKI in hospitalized patients. Clin J Am Soc Nephrol.

[27] Wilson FP, Shashaty M, Testani J, Aqeel I, Borovskiy Y, Ellenberg SS (2015). Automated, electronic alerts for acute kidney injury: a single-blind, parallel-group, randomised controlled trial. Lancet.

[28] Sharma G, Kuo YF, Freeman JL, Zhang DD, Goodwin JS (2010). Outpatient follow-up visit and 30-day emergency department visit and readmission in patients hospitalized for chronic obstructive pulmonary disease. Arch Intern Med.

[29] Hernandez AF, Greiner MA, Fonarow GC, Hammill BG, Heidenreich PA, Yancy CW (2010). Relationship between early physician follow-up and 30-day readmission among Medicare beneficiaries hospitalized for heart failure. JAMA.

[30] US Centers for Medicare and Medicaid Services: Readmissions Reduction Program. http://www.cms.gov/Medicare/Medicare-Fee-for-Service-Payment/AcuteInpatientPPS/Readmissions-Reduction-Program.html. Accessed October 1 2014.

[31] Berwick DM (2002). A user’s manual for the IOM’s’Quality Chasm’ report. Health Aff.

[32] Allen AS, Forman JP, Orav EJ, Bates DW, Denker BM, Sequist TD (2011). Primary care management of chronic kidney disease. J Gen Intern Med.

[33] Avorn J, Bohn RL, Levy E, Levin R, Owen WF, Winkelmayer WC (2002). Nephrologist care and mortality in patients with chronic renal insufficiency. Arch Intern Med.

[34] Coleman EA, Smith JD, Raha D, Min SJ (2005). Posthospital medication discrepancies: prevalence and contributing factors. Arch Intern Med.

[35] Tam VC, Knowles SR, Cornish PL, Fine N, Marchesano R, Etchells EE (2005). Frequency, type and clinical importance of medication history errors at admission to hospital: a systematic review. CMAJ.

[36] Bell CM, Brener SS, Gunraj N, Huo C, Bierman AS, Scales DC (2011). Association of ICU or hospital admission with unintentional discontinuation of medications for chronic diseases. JAMA.

[37] Dasgupta K, Quinn RR, Zarnke KB, Rabi DM, Ravani P, Daskalopoulou SS (2013). The 2014 Canadian Hypertension Education Program Recommendations for Blood Pressure Measurement, Diagnosis, Assessment of Risk, Prevention, and Treatment of Hypertension. Can J Cardiol.

[38] Canadian Diabetes Association Clinical Practice Guidelines Expert Committee (2013). Canadian Diabetes Association 2013 Clinical Practice Guidelines for the Prevention and Management of Diabetes in Canada. Can J Diabetes.

[39] Kidney Disease: Improving Global Outcomes (KDIGO) CKD Work Group (2013). KDIGO 2012 Clinical Practice Guideline for the Evaluation and Management of Chronic Kidney Disease. Kidney Int Suppl.

[40] Kidney Disease: Improving Global Outcomes (KDIGO) Lipid Work Group (2013). KDIGO Clinical Practice Guideline for Lipid Management in Chronic Kidney Disease. Kidney Int Suppl.

[41] Tseng CL, Kern EF, Miller DR, Tiwari A, Maney M, Rajan M (2008). Survival benefit of nephrologic care in patients with diabetes mellitus and chronic kidney disease. Arch Intern Med.

[42] Shah BR, Hux JE, Laupacis A, Mdcm BZ, Austin PC, van Walraven C (2005). Diabetic patients with prior specialist care have better glycaemic control than those with prior primary care. J Eval Clin Pract.

[43] Weber C, Beaulieu M, Djurdjev O, Er L, Taylor P, Ignaszewski A (2012). Towards rational approaches of health care utilization in complex patients: an exploratory randomized trial comparing a novel combined clinic to multiple specialty clinics in patients with renal disease-cardiovascular disease-diabetes. Nephrol Dial Transplant.

[44] Butler CC, Simpson SA, Hood K, Cohen D, Pickles T, Spanou C (2013). Training practitioners to deliver opportunistic multiple behaviour change counselling in primary care: a cluster randomised trial. BMJ.

[45] Akcan-Arikan A, Zappitelli M, Loftis LL, Washburn KK, Jefferson LS, Goldstein SL (2007). Modified RIFLE criteria in critically ill children with acute kidney injury. Kidney Int.

[46] Blinder JJ, Goldstein SL, Lee VV, Baycroft A, Fraser CD, Nelson D (2012). Congenital heart surgery in infants: effects of acute kidney injury on outcomes. J Thorac Cardiovasc Surg.

[47] Slater MB, Anand V, Uleryk EM, Parshuram CS (2012). A systematic review of RIFLE criteria in children, and its application and association with measures of mortality and morbidity. Kidney Int.

[48] Zappitelli M, Parvex P, Joseph L, Paradis G, Grey V, Lau S (2006). Derivation and validation of cystatin C-based prediction equations for GFR in children. Am J Kidney Dis.

[49] Douglas SL, Daly BJ, Kelley CG, O’Toole E, Montenegro H (2007). Chronically critically ill patients: health-related quality of life and resource use after a disease management intervention. Am J Crit Care.

[50] Cuthbertson BH, Rattray J, Campbell MK, Gager M, Roughton S, Smith A (2009). The PRaCTICaL study of nurse led, intensive care follow-up programmes for improving long term outcomes from critical illness: a pragmatic randomised controlled trial. BMJ.

[51] National Quality Forum. http://www.qualityforum.org/Measures_Reports_Tools.aspx. Accessed July 25, 2015.

[52] Craig P, Dieppe P, Macintyre S, Michie S, Nazareth I, Petticrew M (2008). Developing and evaluating complex interventions: the new Medical Research Council guidance. BMJ.

[53] Campbell M, Elbourne D (2004). Group ADftC. CONSORT statement: Extension to cluster randomised trials. BMJ.

[54] Palevsky PM, Molitoris BA, Okusa MD, Levin A, Waikar SS, Wald R (2012). Design of clinical trials in acute kidney injury: report from an NIDDK workshop on trial methodology. Clin J Am Soc Nephrol.

[55] Liem YS, Bosch JL, Hunink MG (2008). Preference-based quality of life of patients on renal replacement therapy: a systematic review and meta-analysis. Value Health.

