# Supplementary material for: Ambulatory care after acute kidney injury: an opportunity to improve patient outcomes
Source: Can J Kidney Health Dis. 2015 Oct 6;2:36. doi: 10.1186/s40697-015-0071-8 (PMC4595050; doi:10.1186/s40697-015-0071-8)
Supplement: Additional file 1: Figure S1. — Acute Kidney Injury Follow-up Clinic referral form (adult clinic). Legend: AKI = acute kidney injury, CRRT = continuous renal replacement therapy, CV = cardiovascular, ICU = intensive care unit, IHD = intermittent hemodialysis, NP = nurse practitioner, SLED = sustained low efficiency dialysis (PDF 280 kb) [file 40697_2015_71_MOESM1_ESM.pdf]

St. Michael's
Inspired Care.
Inspiring Science.

# Acute Kidney Injury (AKI) Follow-up Clinic Referral Form

Date of Referral: ___________________ Staff MD/NP: _________________________

Referral Source:

- ❑ Internal medicine
- ❑ CV surgery
- ❑ Cardiology
- ❑ Nephrology
- ❑ Other: ____________________________________

Dialysis required: ❑ Yes ❑ No
If Yes: ❑ CRRT ❑ SLED ❑ IHD ❑ Unknown

ICU stay: ❑ Yes ❑ No

Nephrology consults involved: ❑ Yes ❑ No

Does patient have an outpatient nephrologist: ❑ Yes ❑ No

Baseline creatinine: ______________________ or ❑ unknown

Expected date of hospital discharge: ______________________________________________________

Additional details:

_______________________________________________________________________________________

_______________________________________________________________________________________

_______________________________________________________________________________________

Fax consultation request to—416-867-3709
AKI clinic secretary—416-867-7460 (ext. 8209)

All AKI clinics are held on 61 Queen Street, 9th floor. The target appointment date is within 30 days of hospital discharge, unless otherwise requested
