# Supplementary material for: Ambulatory care after acute kidney injury: an opportunity to improve patient outcomes
Source: Can J Kidney Health Dis. 2015 Oct 6;2:36. doi: 10.1186/s40697-015-0071-8 (PMC4595050; doi:10.1186/s40697-015-0071-8)
Supplement: Additional file 2: Figure S2. — Advertisement poster for hospital wards (adult clinic). Legend: AKI = acute kidney injury, CKD = chronic kidney disease, eGFR = estimated glomerular filtration rate, HUS = hemolytic-uremic syndrome, TTP = thrombotic thrombocytopenic purpura (DOCX 43 kb) [file 40697_2015_71_MOESM2_ESM.docx]

**The Acute Kidney Injury (AKI) Follow-up Clinic at St. Michael’s Hospital**

**Did you know???**

🡪AKI survivors have a *40% increased risk of dying* in the 2 years after the initial hospitalization

🡪AKI is associated with the *development of new or accelerated chronic kidney disease*

🡪the *association of* *AKI with long-term mortality* is present even in patients with rapidly reversible AKI

After an episode of AKI…

**Refer to the AKI Follow-up Clinic**

Fax: 416-867-3709 Tel: 416-867-7460 (ext. 8209)

Email: [akiclinic@smh.ca](mailto:akiclinic@smh.ca)

All patients with an episode of AKI should be considered for post-discharge nephrology care

***Referral criteria (even if partially or completely recovered kidney function):***

- doubling of serum creatinine during hospitalization or from known pre-admission baseline
- creatinine over 354µmol/L during an AKI episode
- requirement for any dialysis to manage AKI

***Exclusion criteria:***

- kidney transplant recipients
- severe pre-existing CKD (baseline eGFR<30mL/min/1.73m^2^)
- diagnosis of glomerulonephritis, vasculitis, HUS/TTP, polycystic kidney disease, myeloma
- palliation as primary goal of care
- patients with previously established nephrology follow-up (including need for dialysis post-discharge)
