# Supplementary material for: Ambulatory care after acute kidney injury: an opportunity to improve patient outcomes
Source: Can J Kidney Health Dis. 2015 Oct 6;2:36. doi: 10.1186/s40697-015-0071-8 (PMC4595050; doi:10.1186/s40697-015-0071-8)
Supplement: Additional file 3: Figure S3. — Standardized assessment form for new patients (adult clinic). Legend: ACEi = angiotensin-converting-enzyme inhibitor, ACR = albumin to creatinine ratio, AKI = acute kidney injury, ARB = angiotensin receptor blocker, ASA = Acetylsalicylic acid, BB = beta-blocker, BP = blood pressure, CHF = congestive heart failure, CKD = chronic kidney disease, DHP = Dihydropyridine, DM2 = diabetes mellitus type 2, eGFR = estimated glomerular filtration rate, LVEF = left ventricular ejection fraction, NSAID = non-steroidal anti-inflammatory drug, PCP = primary care provider (DOCX 20 kb) [file 40697_2015_71_MOESM3_ESM.docx]

**Supplementary Figure 3: Standardized assessment form for new patients**

Thank you for referring your patient to the acute kidney injury (AKI) follow-up clinic. AKI survivors have a 40% increased risk of dying in the 2 years after the initial hospitalization, and AKI is associated with the development of new or accelerated chronic kidney disease. We will see patients in clinic 2-3 times per year, and follow bloodwork quarterly. The objective of the AKI clinic is to reduce the long-term morbidity and mortality of AKI survivors.

**Clinic Impression and Recommendations:**

**CKD stage:** ____________________ Creatinine:_________ eGFR:___________

**BP target:** DM2 = 130/80 ACR over 70mg/mmol = 130/80 All others = 140/90

If DM2 or high ACR🡪First line drug=ACE inhibitor or ARB

Second line drug = DHP calcium channel blocker or thiazide diuretic

**Proteinuria:**______________ Target below 1g per day (ACR under 70mg/mmol)

If proteinuria above target🡪 First line drug=ACE inhibitor or ARB

Second line drug=blood pressure control

**Secondary prevention meds:** [ASA] [Statins] [BB] [ACEi/ARB]

Statins are recommended for most patients with eGFR<60mL/min/1.73m^2^

**Drugs stopped:**____________________________ **Drugs started:**______________________________

Consider stopping all ACE inhibitors or ARBs for 3 months in patients with preserved LVEF

**Procedures ordered:** [Ultrasound] [Echo] [Other]________________________

**Referrals:** [Cardio/CHF] [Endocrine] [Other]________________________

**Sick day counselling** (NSAIDs, diuretics, ACEi/ARB) **Family present:** [Yes] [No]

**Lifestyle counselling** (diet, smoking, exercise) **Communication with PCP:** [Yes] [No]

**Other:**

**Return to Clinic:** [3 months] [6 months] **All patients complete labs every 3 months**
