# Supplementary material for: Ambulatory care after acute kidney injury: an opportunity to improve patient outcomes
Source: Can J Kidney Health Dis. 2015 Oct 6;2:36. doi: 10.1186/s40697-015-0071-8 (PMC4595050; doi:10.1186/s40697-015-0071-8)
Supplement: Additional file 4: Figure S4. — Sick day medication list (adult clinic) (DOCX 19 kb) [file 40697_2015_71_MOESM4_ESM.docx]

**Sick Day Medication Advice**

Some of your routine medications may need to be stopped for a few days if you become sick. This is very important if you experience diarrhea, vomiting, or are unable to eat or drink. The following types of medications should not be taken when you are sick, as they could cause your kidney function to worsen.

Stop blood pressure medications called **ACE-Inhibitors**, these include:

| Benazepril (Lotensin®) | Lisinopril (Prinivil®, Zestril®) |
| --- | --- |
| Captopril (Capoten®) | Perindopril (Coversyl®) |
| Cilazapril (Inhibace®) | Quinapril (Accupril®) |
| Enalapril (Vasotec®) | Ramipril (Altace®) |
| Fosinopril (Monopril®) | Trandopril (Mavik®) |

Stop blood pressure medications called **Angiotensin Receptor Blockers** (ARBs), these include:

| Candesartan (Atacand®) | Olmesartan (Olmetec®) |
| --- | --- |
| Eprosartan (Tevetan®) | Telmisartan (Micardis®) |
| Irbesartan (Avapro®) | Valsartan (Diovan®) |
| Losartan (Cozaar®) |  |

Stop blood pressure medications called **Direct Renin Inhibitors**, these include:

• Aliskiren (Rasilez®)

Stop blood pressure medications called **diuretics or water pills**, these include:

| Amiloride (Midamor®) | Hydrochlorothiazide (Hydrodiuril®) |
| --- | --- |
| Bumetanide (Burinex®) | Indapamide (Lozide®) |
| Chlorthalidone | Metolazone (Zaroxolyn®) |
| Eplerenone (Inspra®) | Spironolactone (Aldactone®) |
| Furosemide (Lasix®) |  |

Stop diabetes medications, these include:

- Metformin (Glucophage®, Glumetza®)

Stop pain medications called **non-steroidal anti-inflammatories (NSAIDs)**, these include:

| Celecoxib (Celebrex®) | Indomethacin (Indocid®) |
| --- | --- |
| Diclofenac (Voltaren®) | Meloxicam (Mobicox®) |
| Fenoprofen (Nalfon®) | Naproxen (Aleve®, Naprosyn®) |
| Ibuprofen (Advil®, Motrin®) |  |

**In fact, you should probably not be taking these pain medications even if you are feeling well because they can cause kidney injury**

You can restart your medications again as soon as you feel better. If you are sick for over a week, contact your family doctor and the AKI clinic.

Always remember to check with your **pharmacist** before using **new, over-the-counter, or herbal medications** to make sure they do not affect the kidney.
