# Supplementary material for: Ambulatory care after acute kidney injury: an opportunity to improve patient outcomes
Source: Can J Kidney Health Dis. 2015 Oct 6;2:36. doi: 10.1186/s40697-015-0071-8 (PMC4595050; doi:10.1186/s40697-015-0071-8)
Supplement: Additional file 5: Figure S5. — Exit pamphlet for patients who graduate from clinic (adult clinic) (DOCX 13 kb) [file 40697_2015_71_MOESM5_ESM.docx]

**Acute Kidney Injury Information Pamphlet**

For Patients who have Graduated from Clinic

**Please give a copy to your family doctor**

**What:** Acute kidney injury is a sudden decline in kidney function. It is a common problem for hospitalized patients.

**Why is this important:** Acute kidney injury can lead to permanent kidney damage, cardiac disease, and/or death. These risks continue even after hospital discharge. Survivors of acute kidney injury have worse long-term outcomes than heart attack and diabetes patients.

**My acute kidney injury episode occurred on:**

**The cause was:**

I was followed in the Acute Kidney Injury Follow-up Clinic at St. Michael’s Hospital. I was discharged from clinic because my eGFR was over 45mL/min/1.73m^2^ and my kidney function remained stable for over 1 year.

**The following steps should be taken in the future to monitor and maintain kidney function:**

- Creatinine check yearly
- Urine albumin:creatinine ratio yearly
- Control of cardiac risk factors
- Blood pressure control
- Avoidance of non-steroidal anti-inflammatory drug (Ibuprofen, Advil, Motrin)

**Please re-refer to the Acute Kidney Injury Follow-up Clinic if:**

- I have another acute kidney injury episode
- Tel: 416-867-7460 (ext. 8209); Fax: 416-867-3709

**Please refer the patient to a General Nephrology clinic if:**

- My eGFR is persistently under 45mL/min/1.73m^2^
- My urine albumin to creatinine raito is persistently over 30mg/mmol

Thank you for allowing the Acute Kidney Injury Follow-up Clinic to participate in your care.

Questions? Contact the Acute Kidney Injury Follow-up Clinic:

Tel: 416-867-7460 (ext. 8209)

Fax: 416-867-3709
