# Supplementary material for: Ambulatory care after acute kidney injury: an opportunity to improve patient outcomes
Source: Can J Kidney Health Dis. 2015 Oct 6;2:36. doi: 10.1186/s40697-015-0071-8 (PMC4595050; doi:10.1186/s40697-015-0071-8)
Supplement: Additional file 6: Figure S6. — Acute Kidney Injury Knowing Note for pediatric patients (DOCX 2488 kb) [file 40697_2015_71_MOESM6_ESM.docx]

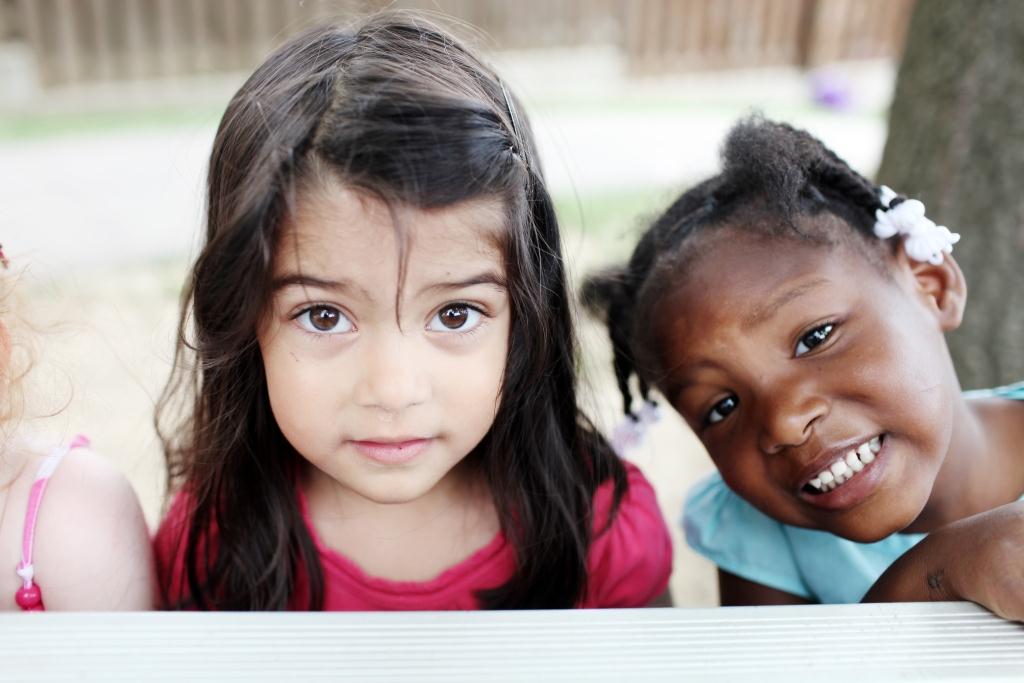

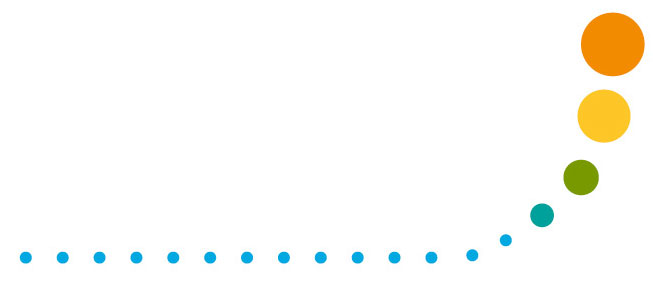

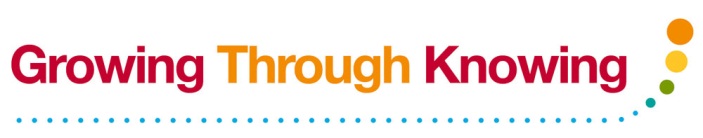

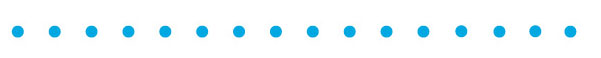

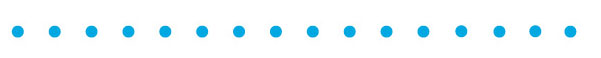


The purpose of this brochure is to provide you with information about acute kidney injury (AKI).

- What is AKI?

- Causes of AKI?
- Management and follow up for AKI.

Questions for my doctor:

________________________________________________________________________________________________________________________________________________________________________________________________________________________________________________________________________________________

**More Information and Resources**

For More Information, please contact:

Contact Information:

Name

Department

Phone

**Acute Kidney Injury**

**(AKI)**

**More information and resources**

For more information, call:

**The Center for**

**Acute Care Nephrology**

**Elizabeth J Rompies, MSN, RN, CPNP**

**Cincinnati Children’s Hospital Medical Center**

**513-803-4408**

**To Schedule: Patti at 513-803-3295**

KNOWING NOTES

**AKI has little to no symptoms**

Simply put, AKI does not hurt. You may not notice any symptoms at all.

It is possible to lose up to 50% of your kidney function before you notice any changes in your body.

- This can be dangerous since you may not see a doctor before your kidneys have been considerably damaged.
- Getting regular follow – up care with your kidney doctor is important to avoid permanent damage to your kidneys (CKD).

Routine care is very important to keep your kidneys healthy and working well.

**How can you tell if my child has AKI?**

The doctor may order the following tests:

- Blood tests
- Urine tests
- Ultrasounds, X-rays

**What is the Treatment for AKI?**

**There is no cure for AKI.**

You will play an important role in keeping your kidneys healthy. Check with the kidney doctor before taking:

- Pain/fever reducers/anti-inflammatory medicines
- Blood pressure lowering drugs
- Decongestants
- Antibiotics
- Herbal products/nutritional supplements

You or your child will need to see the doctor on a regular basis to keep his/her kidneys healthy and prevent CKD.

**When should I call the doctor?**

Check with the kidney doctor if you or your child is:

- Scheduled for surgery
- Prescribed an antibiotic
- Admitted to the hospital with an illness or infection
- Scheduled for a CT scan or MRI

**Important Facts about Acute Kidney Injury (AKI)**

- AKI is a sudden decrease in the function of the kidney.
- Over time, without proper check-ups, this sudden change could lead to a permanent loss of kidney function (Chronic Kidney Disease or “CKD”).
- Seeing the doctor on a regular basis allows for testing of the blood and urine to see how well your kidneys are working.

**What Causes AKI?**

There are many factors that may cause AKI, such as**:**

- Certain medications
  - Medicines used in imaging
  - Pain/fever reducers
  - Medicines used to control blood pressure
- Heart problems and/or surgeries to correct them
- Infections in the blood
- Problems with the liver
- Dehydration, blood loss, burns
