# Supplementary material for: Ambulatory care after acute kidney injury: an opportunity to improve patient outcomes
Source: Can J Kidney Health Dis. 2015 Oct 6;2:36. doi: 10.1186/s40697-015-0071-8 (PMC4595050; doi:10.1186/s40697-015-0071-8)
Supplement: Supplementary file 7 [file 40697_2015_71_MOESM7_ESM.docx]

Medications that May Harm Your Kidneys

Medication Tips for Safe Use

Discuss your Chronic Kidney Disease (CKD) status with the prescribing provider before starting any new medication(s), including oral or intravenous **contrast agents** used in imaging.

Always talk with your provider before trying **herbal products or nutritional supplements**. Some may have dangerous side effects or may interact with other medications you are taking.

Certain **antibiotics** (used to treat infections) may harm your kidneys. Choice and/or dose of an antibiotic may need to change based on your CKD status.

Ask your provider or pharmacist about safe doses or alternatives.

If you have questions about your medications and their risk of kidney harm, please call:

____

Cincinnati Children's

Rev. 6.04.14

RED ZONE = AVOID

Do NOT use these medications unless directed by your Urologist/Nephrologist

Pain / Fever Reducers / Anti-Inflammatory

- Non-steroidal anti-inflammatory drugs (NSAIDs)
  - Ibuprofen (Advil®, Motrin®)
  - Naproxen (Aleve®, Naprosyn)
  - Ketorolac (Toradol®)

Yellow Zone = Caution

These medications treat other health problems your child may have. Discuss with your provider.

Blood Pressure Lowering Drugs

- Captopril (Capoten®)
- Enalapril (Vasotec®)
- Lisinopril (Prinivil® , Zestril®)
- Losartan (Cozaar®)

Decongestants

(in "Multi-Symptom" Cough, Cold and Flu Products)

- Phenylephrine (Sudafed PE ®, Triaminic Cough and Cold, most Dimetapp products)
- Pseudoephedrine (Sudafed ®; combination cough, cold, flu, and allergy products, such as Claritin-D ®)

Anti-Virals

Drink plenty of fluids while taking these medications

- Acyclovir (Zovirax®)
- ValACYclovir (Valtrex®)
- ValGANcyclovir (Valcyte®)
